# Optimizing mesoderm progenitor selection and three-dimensional microniche culture allows highly efficient endothelial differentiation and ischemic tissue repair from human pluripotent stem cells

**DOI:** 10.1186/s13287-016-0455-4

**Published:** 2017-01-23

**Authors:** Fengzhi Zhang, Lin Wang, Yaqian Li, Wei Liu, Fuyu Duan, Rujin Huang, Xi Chen, Sophia Chia-Ning Chang, Yanan Du, Jie Na

**Affiliations:** 1 Center for Stem Cell Biology and Regenerative Medicine, School of Medicine, Tsinghua University, Beijing, 100084 China; 2 Department of Biomedical Engineering, School of Medicine, Collaborative Innovation Center for Diagnosis and Treatment of Infectious Diseases, Tsinghua University, Beijing, 100084 China; 3 Department of Plastic Surgery, Beijing Tsinghua Changgung Hospital, Beijing, 102218 China; 4 School of Medicine, Tsinghua University, Beijing, 100084 China

**Keywords:** Human pluripotent stem cells, Endothelial cells, MESP1, 3D culture, Vascularization

## Abstract

**Background:**

Generation of large quantities of endothelial cells is highly desirable for vascular research, for the treatment of ischemia diseases, and for tissue regeneration. To achieve this goal, we developed a simple, chemically defined culture system to efficiently and rapidly differentiate endothelial cells from human pluripotent stem cells by going through an MESP1 mesoderm progenitor stage.

**Methods:**

Mesp1 is a key transcription factor that regulates the development of early cardiovascular tissue. Using an MESP1-mTomato knock-in reporter human embryonic stem cell line, we compared the gene expression profiles of MESP1^+^ and MESP1^−^ cells and identified new signaling pathways that may promote endothelial differentiation. We also used a 3D scaffold to mimic the in vivo microenvironment to further improve the efficiency of endothelial cell generation. Finally, we performed cell transplantation into a critical limb ischemia mouse model to test the repairing potential of endothelial-primed MESP1^+^ cells.

**Results:**

MESP1^+^ mesoderm progenitors, but not MESP1^−^ cells, have strong endothelial differentiation potential. Global gene expression analysis revealed that transcription factors essential for early endothelial differentiation were enriched in MESP1^+^ cells. Interestingly, MESP1 cells highly expressed Sphingosine-1-phosphate (S1P) receptor and the addition of S1P significantly increased the endothelial differentiation efficiency. Upon seeding in a novel 3D microniche and priming with VEGF and bFGF, MESP1^+^ cells markedly upregulated genes related to vessel development and regeneration. 3D microniches also enabled long-term endothelial differentiation and proliferation from MESP1^+^ cells with minimal medium supplements. Finally, we showed that transplanting a small number of endothelial-primed MESP1^+^ cells in 3D microniches was sufficient to mediate rapid repair of a mouse model of critical limb ischemia.

**Conclusions:**

Our study demonstrates that combining MESP1^+^ mesoderm progenitor cells with tissue-engineered 3D microniche and a chemically defined endothelial induction medium is a promising route to maximizing the production of endothelial cells in vitro and augment their regenerative power in vivo.

**Electronic supplementary material:**

The online version of this article (doi:10.1186/s13287-016-0455-4) contains supplementary material, which is available to authorized users.

## Background

Human pluripotent stem cells (hPSCs) can self-renew indefinitely and differentiate into all types of tissues in the body. Therefore, it can be an unlimited source to provide specialized cells for biomedical research and cell-based therapy. hPSC-derived endothelial cells (ECs) may be used to study angiogenesis and regenerate blood vessels in ischemic diseases [1, 2]. Two methods of endothelial differentiation have been developed. One protocol uses embryoid bodies (EBs) as a differentiation intermediate, followed by EC purification using specific surface antibodies [1, 2]. As the cell types in EBs are highly heterogeneous, the differentiation efficiency of this protocol is relatively low. The second protocol was developed more recently, where, a monolayer culture and sometimes chemically defined conditions are used to achieve directed and more efficient EC differentiation. hPSCs are first induced to form mesoderm, then vascular endothelial growth factor (VEGF) and basic fibroblast growth factor (bFGF) are added to specify the endothelial fate [3–7]. Such monolayer and serum-free protocols can be modified to screen for small molecules that may enhance differentiation and are more compatible with requirements for clinical applications. The reported yields of ECs range from 20 to 80%, depending on the time period of differentiation and whether selection and purification steps were included in the protocol [3–7].

To achieve efficient in vitro differentiation and obtain cells more closely resembling in vivo counterparts, it is important to follow the developmental principle of the desired lineage. During embryo development, a subgroup of extraembryonic mesoderm cells forms primitive endothelial cells in the yolk sac [8]. Mesoderm posterior 1 (Mesp1) is a basic helix–loop–helix (bHLH) transcription factor first expressed in the nascent mesoderm during gastrulation [9, 10]. These cells then migrate laterally and anteriorly and give rise to the heart and great vessels [9, 10]. In addition, Mesp1 progenies also contribute to extraembryonic mesoderm [9]. The fate of this subgroup of cells is less well studied. Studies in mice and zebrafish convincingly demonstrated that Mesp1 represents the earliest marker of cardiovascular progenitors [9, 11, 12]. However, whether MESP1 progenitor cells can be a good source to generate ECs during hPSC in vitro differentiation is unknown.

Previously, we generated an MESP1 reporter human embryonic stem cell (hESC) line with mTomato knocked in just before the stop code of the MESP1 coding sequence (manuscript submitted). In the latter study, we showed that in mouse embryos, Mesp1 progeny cells gave rise to vasculature in the yolk sac of the mouse embryo. In hPSCs, MESP1-mTomato^+^ cells could differentiate to ECs at high efficiency. Moreover, plating MESP1^+^ cells in three-dimensional (3D) microniches significantly improved the EC differentiation efficiency and the long-term survival ability, and it facilitated cell transplantation repair of a critical limb ischemia mouse model. A global gene expression study revealed novel genes and pathways that might regulate EC differentiation and proliferation during the mesoderm induction and endothelial fate specification stages. Our work suggests that following developmental cues and using 3D microniches that resemble the in vivo cellular microenvironment may greatly facilitate directed differentiation from hPSCs and augment the repairing power of transplanted cells.

## Methods

### hPSC culture

H9 hESCs (WiCell Institute Inc., Madison, WI, USA), H9-MESP1-mTomato hESC reporter cell line (generated by LW), and human induced pluripotent stem cells (hiPSCs) (CD34-iPSCs, generated by FD) were routinely maintained on MEF feeders in the hESC medium: KnockOut Dulbecco’s modified Eagle’s medium (DMEM) culture medium supplemented with 20% (vol/vol) KnockOut serum replacement, 1% nonessential amino acids, 1 mM L-GlutaMAX-I, 0.1 mM β-mercaptoethanol, and 8 ng/ml bFGF. They were passaged with 1 mg/ml collagenase IV (Invitrogen, Carlsbad, CA, USA) and seeded onto a 25 cm^2^ flask that had been previously coated with 0.1% gelatin solution (Sigma-Aldrich, St. Louis, MO, USA). To achieve feeder-free culture, hESCs were maintained on Matrigel (BD Biosciences, San Jose, CA, USA)-coated plates (Corning, Corning, NY, USA) in E8 medium (Stemcell Technologies, Vancouver, Canada).

### Generation of MESP1-mTomato knocking-in reporter cell line

The homologous recombination donor vector was composed of the MESP1 left arm, T2A fused with a membrane-bound tdTomato (mTomato), PGK promoter-driving puromycin resistance gene (PGK-Puro), MESP1 right arm, and MC-1 promoter-driving TK gene. H9 cells were electroporated with TALEN and donor vectors by Neon microporator (Invitrogen). After puromycin selection, targeted clones were picked and expanded for characterization. Detailed protocols (design of TALEN pair and synthesis of tandem arrays of TALE repeats) have been described elsewhere (manuscript submitted).

### RNA isolation, Q-PCR and high-throughput RNA sequencing

Total RNA was isolated from undifferentiated hESCs, differentiation day 3 cells, and differentiation day 8 cells on two-dimensional (2D) plates and 3D microniches using RNeasy Plus Mini Kit (Qiagen, Hilden, Germany) and treated with RNase-free DNase. 1 μg RNA of each sample was reverse-transcribed with Superscript III (TransGen Biotech, Beijing, China). Quantitative real-time polymerase chain reaction (Q-PCR) reactions were performed using GoTaq qPCR Master Mix (Promega, Madison, WI, USA) in a CFX96 Real-Time System (Bio-Rad, Hercules, CA, USA) and results were analyzed with the Bio-Rad CFX Manager program. The sample input was normalized against the Ct (critical threshold) value of *GAPDH*. Primer sequences are listed in Additional file 1: Table S1. For high-throughput RNA sequencing, complementary DNA (cDNA) libraries were prepared using the TruSeq™ RNA Sample Preparation kit (Illumina, San Diego, CA, USA) and sequencing was performed at the Biopic sequencing facility of Peking University (http://biopic.pku.edu.cn/english/). The clean reads were mapped to human reference genome (hg19) using BWA software. Cluster analysis of gene expression patterns was performed using Cluster 3.0 and JavaTreeview software. Gene ontology (GO) term enrichment was analyzed using the database for annotation, visualization, and integrated discovery (DAVID) (https://david.ncifcrf.gov). Data are publicly available at the National Center for Biotechnology Information with Gene Expression Omnibus (GEO), accession number GSE79470.

### MESP1^+^ mesoderm progenitor induction

MESP1^+^ mesoderm progenitor induction was performed based on the method described by Cao et al. [13] with some modifications. Briefly, undifferentiated hESCs cultured in E8 medium were digested into single cells by Accutase (EMD Millipore, Billerica, MA, USA) and plated onto Matrigel-coated culture dishes at a density of 6 × 10^4^ cells/cm^2^ in a medium containing DMEM/F12, 1 × B27 supplement (without vitamin A), 1% L-GlutaMAX-I, 50 μg/ml ascorbic acid (Sigma-Aldrich), induced by 25 ng/ml bone morphogenetic protein (BMP)4 and 10 μM GSK3 inhibitor CHIR99021 (Tocris Bioscience, Bristol, UK) for H9 and induced by 2 μM CHIR99021 for CD34-iPSC. Rho-associated coiled-coil kinase (ROCK) inhibitor Y27632 (Tocris Bioscience) (5 μM) was added during cell dissociation and replating for 24 h and it was removed during the medium change. The monolayer-cultivated cells were harvested at differentiation day 3 for further analysis.

### Differentiation of MESP1^+^ cells to endothelial cells in 2D culture system and 3D microniches

Day 3 MESP1^+^ cells were dissociated with Accutase for 10 min and plated onto Matrigel-coated culture dishes at a density of 2 × 10^4^ cells/cm^2^ in a basal medium and stimulated with 25 ng/ml VEGF plus 10 ng/ml bFGF (both Sino Biological, Inc., Beijing, China) for 5 d. For the 3D microniches culture, biodegradable gelatin microcryogels (GMs) were prepared using a microstencil array chip as previously described [14]. Briefly, 6% (wt/vol) gelatin precursor solution was mixed with 0.3% glutaraldehyde, and then 200 μl of precursor solution was pipetted and scraped onto the microstencil array chip. The microstencil array chip underwent cryogelation for 16 h and was then lyophilized for 30 min (Beijing Boyikang Laboratory Instruments, Beijing, China). GMs were then harvested by a PMMA Ejector Pin array fabricated with a desktop 3D milling machine (MDX-40A; Roland DG, Shizuoka-ken, Japan). Before loading the cells, the harvested and dried GMs in the dish were sterilized by an ethylene oxide sterilization system that performed a 12 h degassing step under vacuum after 12 h of gas exposure (AN74j/Anprolene; Anderson Products, Haw River, NC, USA) [15]. A 50 μl MESP1^+^ cell suspension (1 × 10^7^ cells/ml) was subsequently pipetted onto 500 tightly packed GMs and automatically absorbed to hydrate the porous structures and then maintained in a humidified chamber and incubated at 37 °C for 2 h to allow cell attachment. Subsequently, the basal medium supplemented with 25 ng/ml VEGF plus 10 ng/ml bFGF was added for long-term culture.

### FACS analysis

Cells were dissociated into single cells as described before, and resuspended in a FACS washing buffer (PBS with 5% fetal calf serum (FCS) and 2.5 mM EDTA). To perform direct flow cytometry, the samples were then stained for FITC-conjugated CD31 (1:20; Miltenyi Biotec, Bergisch Gladbach, Germany), and FITC-conjugated mouse IgG2a (for CD31, 1:20; Miltenyi Biotec) was used as isotype-matched negative control. Data were collected with a FACSCaliber flow cytometer (BD Biosciences) and analyzed using FlowJo software (Tree Star, Inc., Ashland, OR, USA).

### Immunostaining analysis

Cells were fixed with 4% paraformaldehyde, permeabilized in 0.5% Triton X-100 (Sigma-Aldrich), blocked in 10% normal goat serum (Origene, Rockville, MD, USA), and then incubated with primary antibodies against MESP1 (1:200; Abcam, Cambridge, MA, USA), CD31 (1:100; Santa Cruz Biotechnology, Dallas, TX, USA), VE-cadherin (1:200; Abcam), αSMA (1:200; Abcam), human nuclei antibody (1:100; Abcam) in 4 °C overnight and detected by DyLight 488- or 549-conjugated secondary antibodies (Thermo Fisher Scientific, Waltham, MA, USA). Nuclei were stained with 4′, 6-diamidino-2-phenylindole (DAPI) (Sigma-Aldrich). A Nikon (Tokyo, Japan) Ti-U fluorescence microscope was used for image acquisition.

### hPSC-EC functional studies

The biological function of hPSC-ECs was assessed by DiI-conjugated acetylated low-density lipoprotein (DiI-ac-LDL) uptake and vascular tube formation. For the DiI-ac-LDL uptake assay, hPSC-ECs were incubated with 20 mg/ml of DiI-Ac-LDL (Yeasen Biotech, Hong Kong) at 37 °C for 6 h, washed with PBS, and stained with Hoechst 33258 (Dojindo Kumamoto, Japan). For the vascular tube formation assay, 1 × 10^5^ cells were plated into one well of 24-well plates precoated with Matrigel (BD Biosciences) and incubated at 37 °C for 12 h.

### Mouse model of hind limb ischemia fluorescence imaging

Procedures were performed on female BALB/c nude mice (20–25 g; 8–10 weeks) as previously described [16] with the approval from the Internal Review Board, Laboratory Animal Research Center, Tsinghua University. Unilateral left hind limb ischemia in mice was induced by ligating the proximal end of the femoral artery and its branches using 5-0 silk surgical suture as described in [14]. The overlying skin was then closed by 5-0 silk surgical suture. Cell transplantation was performed after skin closure. 300 medium-soaked GMs (acellular controls) or 300 cell-load GMs (3 × 10^5^ cells) were washed with PBS, then resuspended in 300 μl gelatin solution and intramuscularly injected into three sites of the gracilis muscle around the artery incision using 1 ml syringes with a 23-gauge needle. For free cell injection, 1 × 10^7^ cells were mixed with Matrigel and injected as above.

Blood perfusion analysis was performed after surgery at the indicated days post operation on a home-made fluorescence imaging system [17] in reflectance geometry. Animals were injected with indocyanine green (ICG, 0.1 ml of 100 μg/ml) through the tail vein and time-series fluorescence images were collected for 100 min at intervals of 0.2 s.

### Statistical analysis

Quantitative data are expressed as mean ± standard deviation. The statistical significance was determined using a Student’s *t* test (two-tailed) for two groups or one-way ANOVA for multiple groups. A value of *p* < 0.05 was considered statistically significant.

## Results

### MESP1 reporter marked cardiovascular progenitor cells

**Fig. 1:**
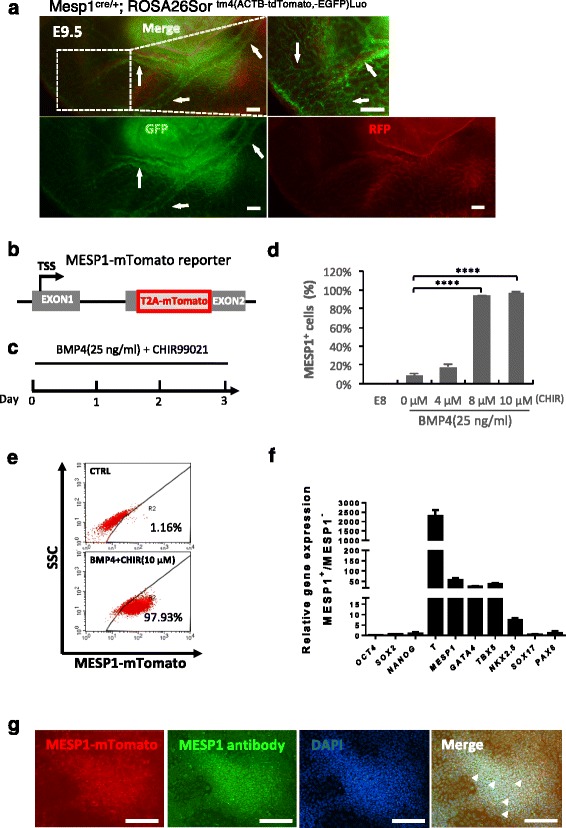
Generation of MESP1^+^ cells from human embryonic stem cells. **a** Mesp1 progeny cells formed yolk sac blood vessels. Images of Mesp1 lineage cells (*green*) on the yolk sac of E9.5 embryos of Mesp1^cre/+^/ROSA26Sor ^tm4(ACTB-tdTomato,-EGFP)Luo^ genetic background. **b** Image showing MESP1-mTomato knock-in reporter cells. **c** Protocol to derive MESP1^+^ cells from hESCs by treatment with BMP4 and CHIR99021. **d** Percentage of MESP1^+^ cells on day 3 under different induction conditions (*n* = 3, ^****^ *p* < 0.0001.). **e** FACS analysis showing, on day 3, that 97.93% of cells turned on MESP1-mTomato after treatment with BMP4 (25 ng/ml) and CHIR (10 μM). **f** Q-PCR analysis showing the downregulation of pluripotency, endoderm and ectoderm marker genes, and upregulation of mesoderm and cardiac markers genes in MESP1^+^ cells vs. MESP^–^ cells (*n* = 3). **g** MESP1-mTomato reporter expression co-localized with anti-MESP1 antibody (*arrowheads*). Scale bars: 100 μm

Mesp1 is a master regulator of early heart development [9, 11, 18]. Using mouse embryos from Mesp1^cre/+^ crossed with ROSA26Sor^tm4(ACTB-tdTomato,-EGFP)Luo^, we found that in addition to the heart, Mesp1 progenies (marked by GFP) also formed yolk sac blood vessels, suggesting that a subgroup of Mesp1 cells may have strong endothelial differentiation potential given the appropriate induction cues (Fig. 1a). To study MESP1 cells during endothelial differentiation in the human system, we generated an MESP1-mTomato reporter hESC line through TALEN-mediated homologous recombination, which enabled us to monitor and purify MESP1-expressing mesoderm progenitor cells (Fig. 1b; manuscript submitted).

MESP1-mTomato reporter cells do not show any fluorescence in undifferentiated hESCs cultured in E8 medium, but strongly turned on red fluorescence in response to BMP4 and increasing concentrations of CHIR99021 (a small molecule inhibitor of GSK3 and activator of canonical Wnt signaling) treatment (Fig. 1c and d). We could obtain nearly 100% MESP1-mTomato-positive cells by treating cells with a combination of BMP4 (25 ng/ml) and glycogen synthase kinase 3 (GSK3) inhibitor CHIR99021 (8–10 μM) in a chemically defined medium consisting of DMEM/F12 and B27(-VitA) supplement (Fig. 1c and d). To obtain a homogeneous population of MESP1^+^ cells, undifferentiated cells cultured in E8 without feeders were dissociated into single cells in the presence of Rho-associated coiled-coil kinase (ROCK) inhibitor Y27632, replated on Matrigel and then treated with BMP4 (25 ng/ml) and CHIR99021 (10 μM). By day 3, FACS analysis showed that most cells were positive for mTomato (Fig. 1e). We also examined the expression of mesodermal and cardiac lineage marker genes by Q-PCR. MESP1^+^ cells significantly upregulated mesoderm transcription factors *T/BRACHYURY* (*T*), *MESP1*, and *GATA4*, as well as early cardiac markers *TBX5* and *NKX2.5.* In contrast, the expression of pluripotency, endoderm and neuroectoderm marker genes, *OCT4*, *SOX2*, *NANOG*, *SOX17*, and *PAX6* were significantly downregulated in MESP1^+^ cells (Fig. 1f). Immunostaining confirmed that mTomato-positive cells co-localized with endogenous MESP1 protein detected by an anti-MESP1 antibody (Fig. 1g). Taken together, MESP1-mTomato reporter cells reflected the expression of endogenous MESP1 and exhibited gene expression typical of early cardiovascular progenitor cells.

**Fig. 2:**
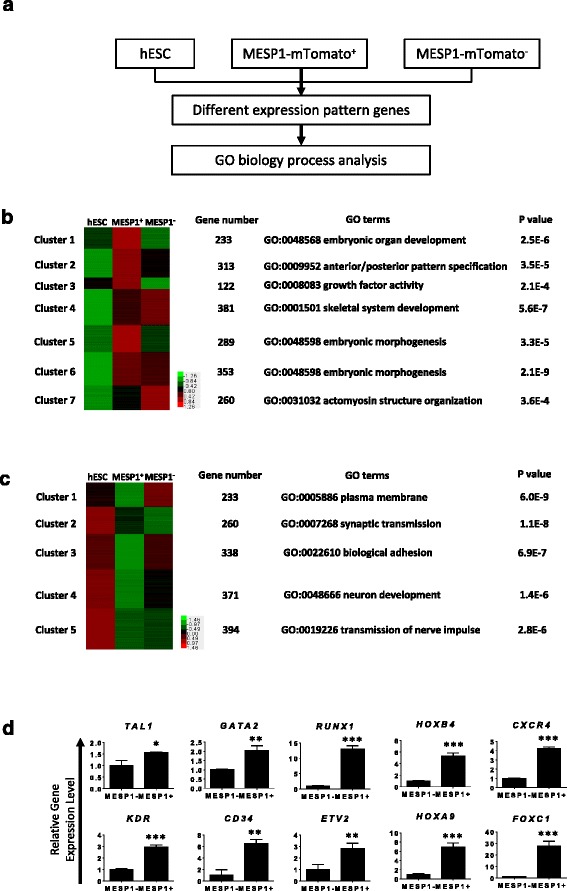
High-throughput RNA sequencing analysis of MESP1-mTomato mesoderm progenitor cells. **a** Flow chart of MESP1-mTomato cell gene expression analysis. **b** and **c** Genes upregulated and downregulated in MESP1-mTomato^+^ cells compared with hESCs (fold change > 1.5). They were divided into different groups based on their FPKM values in hESC, MESP1-mTomato^+^, and MESP1-mTomato^–^ cells. The number of genes in each group, the top GO term, and the enrichment *p* values are listed. **d** Q-PCR validation of key genes enriched in MESP1^+^ cells according to the RNA-seq result (*n* = 3, ^*^ *p* < 0.05, ^**^ *p* < 0.005, ^***^ *p* < 0.001, MESP1^+^ versus MESP1^–^; *t* test)

Next, we performed high-throughput RNA sequencing of MESP1-mTomato positive cells (MESP1^+^) at day 3 of differentiation and compared their gene expression profile with MESP1-mTomato negative cells (MESP1^–^) and undifferentiated hESCs (Fig. 2a). A total of 1951 genes showed a greater than 1.5-fold increase in MESP1-mTomato^+^ versus undifferentiated hESCs, which were grouped into seven clusters based on different dynamic patterns in undifferentiated hESCs, MESP1^+^, and MESP1^–^ cells (Fig. 2b). Gene ontology (GO) analysis showed that clusters 1, 2, 3, and 5 (upregulated in MESP1^+^ compared with undifferentiated hESCs or MESP1^–^) were enriched for genes involved in embryonic organ development, anterior/posterior pattern specification, growth factor activity, and embryonic morphogenesis, respectively, which is in accordance with MESP1 functions during embryo development in vivo (Fig. 2b and Additional file 2: Table S2 and Additional file 3: Table S3). A total of 1596 genes in MESP1^+^ cells showed more than 1.5-fold decrease compared to undifferentiated hESCs and they were divided into five clusters according to their different dynamic patterns (Fig. 2c and Additional file 2: Table S2 and Additional file 3: Table S3). GO analysis showed that clusters 4 and 5 were closely related to neural differentiation, which reflects that the one important aspect of mesoderm induction is to inhibit neural fate [19]. Interestingly, the expression of genes involved in the plasma membrane and biological adhesion obviously decreased. This is in agreement with the mesoderm differentiation process that involves an epithelial-to-mesenchymal transition and dramatic downregulation of cell–cell adhesion and selected extracellular matrix (ECM) genes [18]. Genes important for EC differentiation such as *TAL1, GATA2*, *RUNX1*, *HOXB4*, *CXCR4*, *KDR*, *CD34*, *ETV2*, *HOXA9*, and *FOXC1* were among the most significantly upregulated genes in MESP1-mTomato^+^ cells, as confirmed by Q-PCR analysis (Fig. 2d).

### Sphingosine-1-phosphate significantly enhanced CD31 endothelial differentiation

**Fig. 3:**
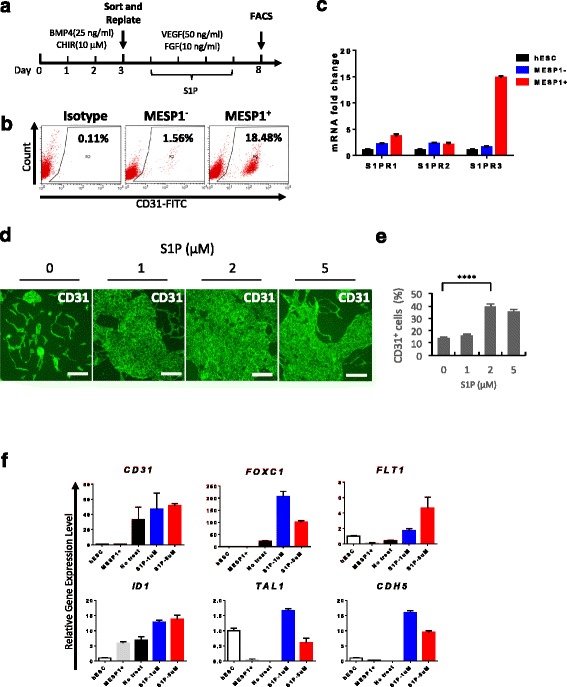
S1P greatly enhanced CD31 expression and upregulated endothelial marker genes expression. **a** Protocol for endothelial differentiation. **b** MESP1^+^ cells have significantly greater ability to differentiate to endothelial cells compared with MESP1^–^ cells. **c** Q-PCR analysis of S1P receptor expression in undifferentiated hESC, differentiation day 3 MESP1-mTomato^–^ (MESP1^–^) and MESP1-mTomato^+^ cells (MESP1^+^). **d** Immunostaining analysis showing that S1P treatment greatly enhanced the generation of CD31^+^ cells on differentiation day 8. Scale bars: 100 μm. **e** Quantification of FACS analysis of CD31^+^ cells percentage after S1P treatment on differentiation day 8 (*n* = 3, ^****^ *p* < 0.0001). **f** Q-PCR analysis showing S1P treatment significantly upregulated endothelial marker genes expression (*n* = 3)

To test whether MESP1-mTomato^+^ cells have stronger endothelial differentiation potential, we used a monolayer, serum-free, and chemically defined differentiation system as shown in Fig. 3a. Sorted MESP1^+^ and MESP1^–^ were replated on Matrigel, and VEGF (50 ng/ml) and bFGF (10 ng/ml) were added. After 5 days, cells were harvested for FACS analysis of endothelial cell surface marker CD31 expression. Some 18.5% of MESP1^+^ sorted cells expressed CD31 compared to only 1.5% from MESP1^–^ cells (Fig. 3b). This result suggested that, as in the embryo, MESP1^+^ cells indeed had stronger endothelial differentiation potential. From our RNA-seq data, we found that Sphingosine-1-phosphate receptor 3 (S1PR3), but not S1PR1 and S1PR2, was significantly upregulated in MESP1^+^ cells. Q-PCR analysis confirmed the expression patterns of S1PR1, S1PR2, and S1PR3. S1PR3 was nearly 15-fold higher in MESP1^+^ cells compared with hESCs and MESP1^–^ (Fig. 3c). We next wanted to determine whether Sphingosine-1-phosphate (S1P) treatment could also affect hPSC endothelial differentiation. S1P was added during day 3–8 of differentiation, together with VEGF and bFGF. Immunostaining and FACS analysis showed that S1P greatly enhanced CD31 expression. Without S1P, CD31 was detected on 13.5% of cells, while after treatment with 2 μM of S1P, 41.6% of cells expressed CD31 (Fig. 3d and e). In addition, Q-PCR analysis showed that S1P treatment significantly upregulated endothelial marker genes, including *TAL1*, *CDH5*, *ID1*, *FLT1*, *FOXC1*, and *CD31* (Fig. 3f). A similar result was obtained in human iPSC (Additional file 4: Figure S1). Thus, optimizing the MESP1^+^ population percentage during the mesoderm induction stage and co-stimulation with S1P during the EC generation stage could markedly boost the CD31^+^ EC differentiation efficiency.

### Microniches dramatically improved the endothelial differentiation efficiency and cell survival

**Fig. 4:**
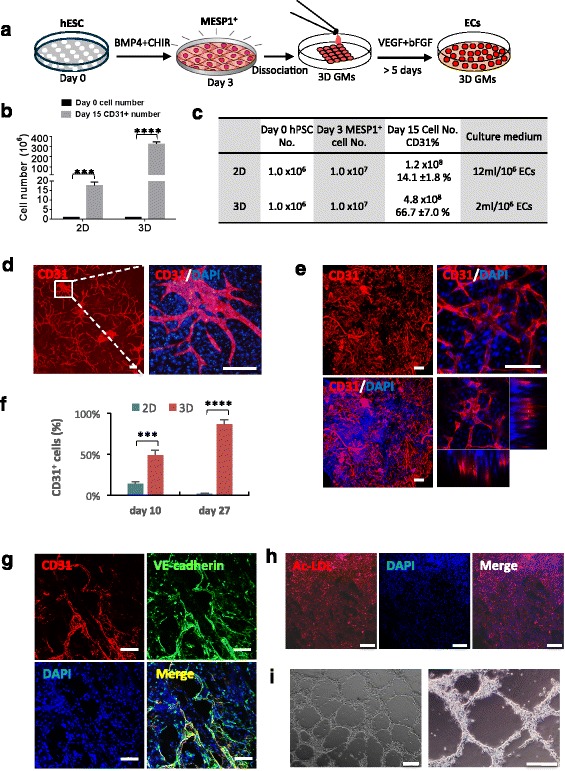
3D microniche significantly improved endothelial differentiation efficiency and helped to maintain long-term cell survival. **a** Protocol for endothelial differentiation from hESC in 3D microniches. **b** Bar graph quantification of CD31+ cell number obtained from 2D and 3D cultures on day 15. **c** Cell numbers at different times and culture medium used in 2D and 3D are listed in the table (*n* = 3, ^***^ *p* < 0.001). **d** Immunostaining showing CD31 expression in 2D condition on differentiation day 10. Scale bars: 100 μm. **e** Immunostaining showing CD31 expression in 3D microniches on differentiation day 10 visualized by multiphoton microscopy. Scale bars: 100 μm. **f** Percentage of CD31^+^ cells during endothelial differentiation under 2D condition and 3D microniches on day 10 and day 27 (*n* = 3, ^***^ *p* < 0.001). **g** ECs differentiated from hiPSCs were immunostained with VE-cadherin and CD31 antibodies. Scale bars: 100 μm. **h** and **i** ECs differentiated from hiPSCs in 3D GMs were digested out and tested in DiI-Ac-LDL uptake and tube formation assay. Scale bars: 100 μm

In vivo vasculogenesis and angiogenesis occur in 3D microenvironments. Next, we tested whether a 3D scaffold could improve endothelial differentiation from MESP1^+^ progenitor cells. To this end, we employed a biodegradable 3D microscale scaffold, which was fabricated by cryogelation of gelatin, and hence was called gelatin microcryogels (GMs) [14]. These 3D GMs had an interconnected macroporous structure with pore sizes in the range of 30–80 μm. They had been shown to be particularly suitable for human mesenchymal stem cell (hMSC) growth [14]. We developed a simple differentiation as depicted in Fig. 4a. Undifferentiated hESCs were dissociated into single cells and cultured in E8 medium to about 30% confluency. Then BMP4 and CHIR99021 were added for 3 days to induce a high percentage of MESP1-mTomato^+^ cells. MESP1^+^ cells were then digested to single cell suspension and loaded onto 500 tightly packed 3D GMs and incubated at 37 °C for 2 hours, during which time cells were absorbed into GMs spontaneously. Afterwards, differentiation medium supplemented with VEGF and bFGF was added for more than 5 days (Fig. 4a). Culturing in 3D GMs significantly promoted cell proliferation, from identical plating hESC numbers (1 × 10^6^ cells), after 15 days of differentiation, the 3D GM group contained an average of 4.8 × 10^8^ cells, while the conventional 2D culture group had only 1.2 × 10^8^ cells (Fig. 4b and c). Moreover, 3D GM differentiation required less frequent medium changes, only once every 3 days, compared to daily medium changes in 2D differentiation during a 12-day window. The quantities of VEGF and bFGF growth factors used were much smaller (Fig. 4c). Based on our calculation, about 320 million ECs could be obtained from one million input hESCs after 15 days of differentiation in 3D, while only 17 million ECs could be obtained in 2D culture during the same time window. Thus, the 3D GM culture appeared to be significantly more cost-effective. Immunostaining revealed that cells formed a tubular network strongly positive for CD31 both in 2D and 3D (Fig. 4d and e). FACS analysis showed that, on differentiation day 10, the 3D GMs contained 49.1 ± 5.9% CD31^+^ cells while the 2D culture only had 14.1 ± 1.8% CD31^+^ cells. By day 27, 86.7 ± 5.0% of cells in 3D GMs were CD31^+^, while only 1.9 ± 0.5% of cells on the 2D surface were CD31^+^ positive owing to overgrowth and cell death (Fig. 4f). Additional experiments using human iPSCs showed similar results (Additional file 5: Figure S2). We also performed tube formation and Ac-LDL absorption assays using cells derived from hESC and iPSC through the MESP1^+^ progenitor stage. ECs digested out from 3D GMs after 12 days strongly expressed CD31 and VE-cadherin (Fig. 4g). They took up Ac-LDL and could organize into a tubular network typical of ECs (Fig. 4h and i). Thus, 3D GMs appeared to significantly boost long-term cell survival of ECs differentiated from MESP1^+^ cells.

### Global gene expression analysis of MESP1^+^ cells endothelial differentiation in 2D and 3D

**Fig. 5:**
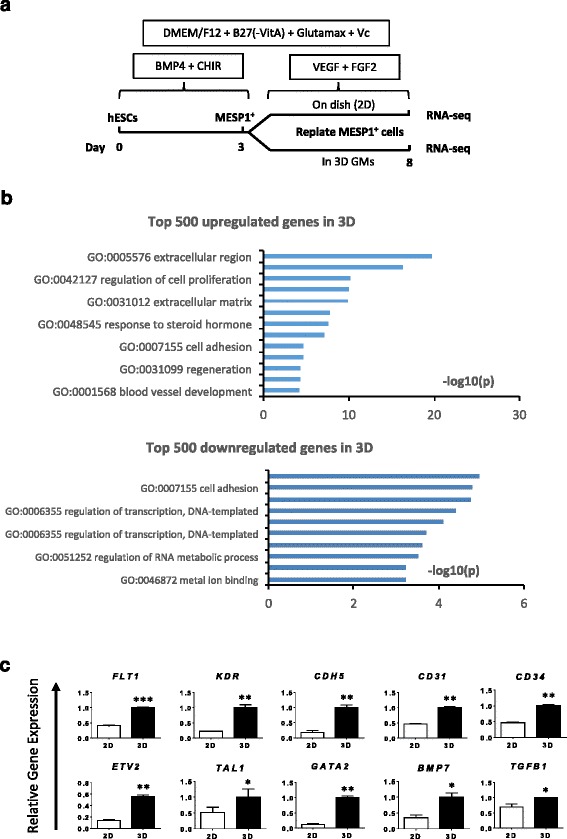
High-throughput RNA sequencing data analysis of MESP1^+^ cells differentiated in 2D and 3D for 5 days. **a** Protocol for endothelial differentiation and RNA-seq. **b** Gene ontology analysis of top 500 upregulated genes and 500 downregulated genes in 3D GMs. **c** Q-PCR validation of representative endothelial marker genes enriched in 3D GM differentiation (*n* = 3, ^*^ *p* < 0.05, ^**^ *p* < 0.005, ^***^ *p* < 0.001, 3D versus 2D; *t* test)

To investigate how the 3D microenvironment influences gene expression, we performed high-throughput RNA sequencing of MESP1^+^ cells differentiated in 2D and 3D, respectively, on day 8, as shown in Fig. 5a. A total of 2102 genes were upregulated more than 1.5-fold in 3D. Among the top 500 genes upregulated in 3D, GO analysis showed a significant enrichment (*p* ≤ 0.01) of the extracellular region, cell proliferation, cell adhesion, regeneration, and vessel development genes (Fig. 5b and Additional file 6: Table S4). Meanwhile, the top 500 downregulated genes in 3D were associated with the regulation of transcription, RNA metabolism, and metal-ion binding (Fig. 5b and Additional file 6: Table S4). Heat maps and clusters of each GO class of upregulated genes in 3D were generated (Additional file 5: Figure S3). Significantly upregulated and downregulated genes in each GO class are listed in Additional file 6: Table S4. We performed Q-PCR analysis to validate representative genes upregulated in 3D, as shown Fig. 5c. Endothelial lineage surface markers *FLT1*, *KDR*, *CDH5*, *CD31*, and *CD34*, growth factors *BMP7* and *TGFB1*, and transcription factors *ETV2*, *TAL1*, and *GATA2* all exhibited enhanced expression of a selection of the genes upregulated in the RNA-seq data (Fig. 5c). These results suggested that a 3D microenvironment might enhance EC differentiation and survival from multiple aspects such as enrichment of secreted growth factors, remodeling of cell adhesion and extracellular matrix, and reorganization of the cytoskeleton, which directly or indirectly affect gene expression.

### MESP1 progenitor cell-derived ECs enhanced vascular repair of critical limb ischemia

**Fig. 6:**
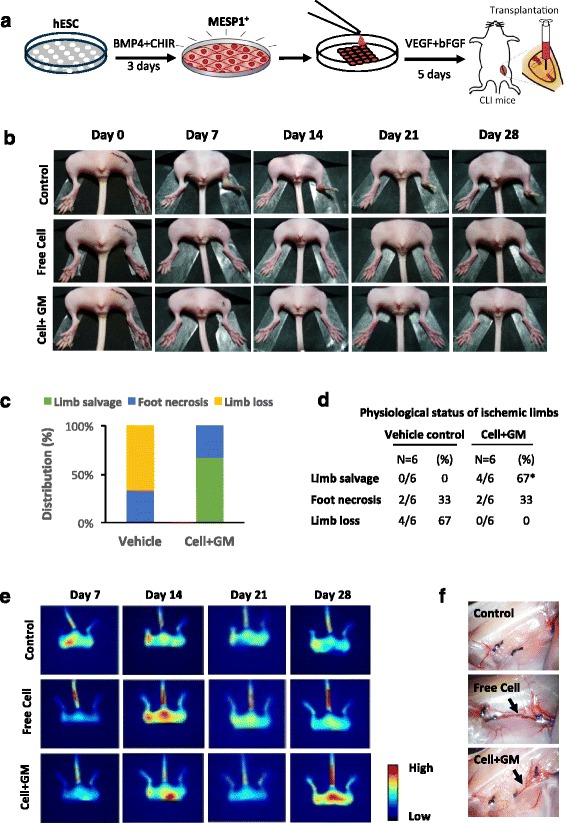
Endothelial induced MESP1 progenitor cells enhanced vascular repair in critical limb ischemia mouse model. **a** Protocol of endothelial cell induction and transplantation in 3D GMs. **b** Representative images of the hindlimb from control, free cell (10^7^), and cell (3 × 10^5^) induced within microcryogels (Cell + GM) transplanted mice. Dates are indicated on the top. (*n* = 6 in each group). **c** Stacked bar graph showing the percentage distribution of the physiological status of treated ischemic limbs on day 28 post-implantation of vehicle and Cell + GM. **d** The table represents the physiological status of the ischemic limb on day 28 post-implantation of vehicle and Cell + GM. Values represent the percentage of limb salvage, necrosis, or loss. Parametric *X* ^2^ test: ^*^ *p* < 0.05. **e** Monitoring blood perfusion in ischemic hindlimb with fluorescence imaging. **f** Photographs showing new blood vessel formation across ligation sites of the ischemic hindlimb 28 days after treatment

To evaluate the therapeutic potential of ECs derived from MESP1^+^ cells, we employed a critical limb ischemia (CLI) mouse model where the ischemia was induced by ligation of the left femoral artery. After surgery, GMs alone (control), MESP1^+^ cells induced with VEGF + bFGF for 5 days (1 × 10^7^ cells) or 3 × 10^5^ MESP1^+^ cells loaded in 3D GMs and induced with VEGF + bFGF for 5 days (Cell + GM) were injected into the ligation site (Fig. 6a). The therapeutic efficacy of cells was examined by evaluating the appearance and physiological status of ischemic limbs at 0, 7, 14, 21, and 28 day after the surgery. Both 1 × 10^7^ cells alone (*n* = 6) and 3 × 10^5^ cells loaded in 3D GMs (Cell + GM) salvaged the ischemia limb within 7 days, while no limb salvage was observed in GM only control group (*n* = 6) after 28 days (Fig. 6b). The Cell + GM combination significantly reduced the number of cells needed to salvage the CLI, since transplanting 3 × 10^5^ cells alone failed to salvage the ischemic hindlimb (data not shown). A total of 67% of mice completely recovered their legs in the Cell + GM group and none of them suffered from limb loss (Fig. 6c and d). In addition, the blood flow was significantly improved by Cell + GM or a high dose (10^7^) of free cells (Fig. 6e). Visual inspection revealed that more blood vessels formed across the ligation site in Cell + GM and high-dose (10^7^) free cell-transplanted CLI mice by day 28 (Fig. 6f). We also confirmed that MESP1^+^ cells do not have tumorigenicity. Undifferentiated MESP1-mTomato reporter cells formed teratoma after subcutaneous injection to the groin region of immune-deficient mice, while differentiated MESP1^+^ cells did not form any teratoma after 8 weeks (Additional file 5: Figure S4). Thus, MESP1 progenies transplanted in 3D GMs can form functional blood vessels in vivo and salvage the CLI mice model.

**Fig. 7:**
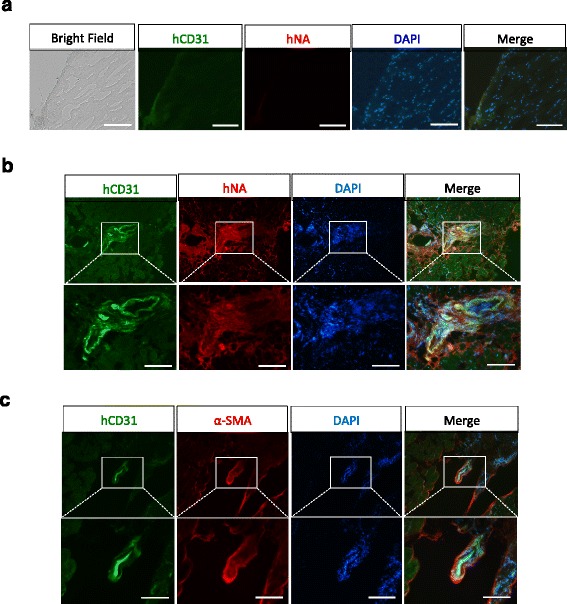
Endothelial induced MESP1 progenitor cells differentiated into blood vessels in critical limb ischemia mouse. **a** Frozen sections from control- treated ischemic hindlimb, no human CD31 (hCD31) and human nuclei (hNA) can be seen on day 28. Scale bar: 100 μm. **b** and **c** Frozen sections from Cell + GM transplanted hindlimb showed clear hCD31 and hNA staining (**b**), and hCD31 and α-SMA co-localization (**c**) at day 28. Scale bars: 100 μm

To check whether injected human cells formed blood vessels in CLI mice, we performed immunostaining of a frozen tissue section using anti-human CD31 antibody and anti-human nuclei antibody. As shown in Fig. 7, no signal was detectable in tissues from GM (Control) transplanted mice (Fig. 7a), while numerous blood vessels were labeled with human CD31 and human-specific nuclei antibodies in tissue sections from cells or Cell + GM transplanted mice, indicating that MESP1^+^ cell-derived ECs had formed human blood vessels in CLI mice, which are responsible for limb salvation (Fig. 7b and c). Thus MESP1^+^ cell-derived ECs could be a potentially useful cell source for blood vessel regeneration and the treatment of ischemic diseases.

## Discussion

In this study, we found that by optimizing the mesoderm induction stage using MESP1-mTomato reporter cells, we can significantly improve the efficiency of EC generation from hESCs and iPSCs. Previous reports mainly focused on vital functions of Mesp1 during heart development, since it is considered the earliest marker of cardiovascular development in vertebrates (Bondue and Blanpain, [11]). Our Mesp1^cre/+^/ROSA26Sor^tm4(ACTB-tdTomato,-EGFP)Luo^ lineage tracing experiments in mice clearly showed that Mesp1 progenies contributed extensively to yolk sac blood vessels, suggesting that Mesp1 cells are also very amenable to taking an endothelial fate given the right induction signal. This is further supported by our RNA-seq results: almost all key regulators of endothelial differentiation were upregulated in MESP1-mTomato^+^ cells, such as *ETV2*, *TAL1*, *GATA2*, *HOXA9*, *FOXC1*, *KDR*, and *CXCR4*. Thus, by monitoring MESP1-mTomato reporter, when MESP1^+^ cells were enriched to a high percentage by adjusting the concentration of BMP4 and GSK3 inhibitor CHIR99021, we can obtain the highest EC formation efficiency. In our hands, 1 hPSC can generate 10 MESP1 positive cells and subsequently 325.34 ± 21.87 ECs after 15 days of induced differentiation. Similar results can be obtained from human iPSCs and other hESC lines, and thus the EC differentiation efficiency positively correlated with the MESP1 expression level during the mesoderm induction stage. Our protocol is also very simple. We used dissociated single hPSCs cultured in E8 medium as the starting population, followed by mesoderm induction by only BMP4 and CHIR99021 for 3 days. Then cells can be replated on a 2D surface or in a 3D scaffold for EC induction and expansion. This protocol is cost-effective and can be modified for large-scale production.

Our RNA-seq data of MESP1^+^ cells provided a valuable resource to uncover signaling pathways that may regulate EC differentiation. We found that S1PR3 is specifically upregulated on MESP1^+^ cells. S1P is a lysophospholipid mediator found in the blood which has been shown to participate in a wide range of biological responses, including stimulation of cell proliferation, inhibition of apoptosis and regulation of cell shapes and motility [20]. Biological activities of S1P are mediated through the S1P receptors, a family of G protein-coupled cell surface receptors. S1PR1, 2, and 3 triple knock-out mice displayed abnormal bleeding and died early during embryogenesis [21]. Mutant embryos had an immature vascular network and the authors concluded that although S1P receptors were not required for endothelial fate specification, they play important roles during angiogenesis [21]. Adding S1P combined with VEGF and bFGF during stage II of the differentiation significantly upregulated many key factors that can promote EC formation, such as *TAL1*, *CDH5*, *ID1*, *FLT1*, *FOXC1*, and *ETV2*, and, as expected, markedly enhanced CD31^+^ EC generation.

In this study, we also found that growing MESP1^+^ cells in 3D GMs can further boost the EC differentiation efficiency. Moreover, 3D GMs can serve as an enrichment platform, since during long-term culture ECs survived better and 85% of cells were strongly CD31 positive after about 4 weeks in 3D GMs. Another advantage of using a 3D GM culture is that the medium did not turn yellow as fast as the medium used for our 2D cultures, despite rapid cell proliferation. Hence, we only needed to change the medium every 3 days compared to daily changes for the 2D culture. Thus the amounts of VEGF and bFGF required for EC differentiation and maintenance were much smaller for our 3D culture. The RNA-seq results revealed that many genes related to ECM, cell adhesion, and blood vessel development were significantly upregulated in 3D. The 3D GMs used in our experiments were fabricated by cryogelation of gelatin. It had an interconnected macroporous structure with pore sizes in the range of 30–80 μm and a high ratio of porosity [14]. These 3D GMs have been shown to attract deposition of ECM proteins and angiogenic growth factors from human mesenchymal stem cells [14]. Endothelial matrix proteins have been shown to regulate EC sprouting, proliferation, and survival [22]. In our study, multiphoton microscopy demonstrated that 3D GM is ideal for the formation of a tubular endothelial network. Thus, the unique characteristics of 3D GMs may promote EC differentiation, proliferation, and long-term survival through both enrichment and stabilization of matrix proteins and angiogenic cytokines and remodeling of cell–matrix and cell–cell adhesion.

3D GM can also serve as a protective niche during cell transplantation. Previously, adult human mesenchymal stem cells were loaded into the same type of 3D GMs and injected into the CLI mouse model. Limb salvation was achieved with as few as 1 × 10^5^ cells [14]. In our case, 67% of mice completely recovered their hind limbs with significantly improved blood flow when only 3 × 10^5^ MESP1^+^ cells were loaded into GMs. Clear revascularization can be seen across the ligation site in either Cell + GM transplanted or 1 × 10^7^ free cell transplanted, but not in 3 × 10^5^ free cell transplanted CLI mice by day 28. It is likely that 3D GM prevented cell loss during injection and after transplantation to the ischemic site in the animal. Interestingly, endothelial primed MESP1^+^ cells formed human blood vessels at the ligation site. Our results suggest that endothelial-primed MESP1 mesoderm progenitor cells combined with 3D GM could be an ideal approach to revasculate ischemic tissues.

## Conclusions

In summary, using MESP1-mTomato reporter cells, we showed that MESP1^+^ mesoderm progenitor cells could be induced to form EC at high efficiency. We also discovered that S1P signaling dramatically enhanced EC formation. Combining these findings with a tissue-engineering approach, we developed a simple, fast, cost-effective, and chemically defined protocol to obtain a high percentage of ECs in biodegradable 3D scaffolds in vitro and demonstrated their superior therapeutic efficacy in treating a CLI mouse model. Our study has opened a new route for cell-based therapy, where patient-specific iPSCs can be converted into MESP1^+^ mesoderm progenitors, then primed toward endothelial fate to treat severe ischemia diseases.

## Additional files

Additional file 1: Table S1. Primers used in this study for gene expression analysis. (DOCX 25 kb)
Additional file 4: Figure S1. Differentiation of hiPSCs to endothelial cells through an MESP1^+^ mesoderm progenitor stage. (a) FACS analysis showing 95.42% of cells stained positive for MESP1^+^ antibody after treating with CHIR99021 (2 μM) for 3 days. (b) Q-PCR analysis showing the downregulation of pluripotency, endoderm and ectoderm marker gene, and the upregulation of mesoderm and cardiac marker genes in MESP1^+^ cells vs. MESP1^-^ cells (*n* = 3). (c) Q-PCR analysis of S1P receptor expression in undifferentiated iPSCs in differentiation day 3 MESP1^+^ and MESP1^-^ cells (*n* = 3). (d) FACS analysis showing S1P treatment greatly enhanced the generation of CD31^+^ cells on differentiation day 12. (PDF 175 kb)
Additional file 5: Figure S2. 3D microniche dramatically improved endothelial differentiation efficiency from hiPSCs. (a and b) Immunostaining showing CD31 expression in 2D (a) and 3D GM (b) on differentiated day 12 respectively. Scale bars: 100 μm. (c) Percentage of CD31^+^ cells on differentiated day 12 in 2D and 3D GM (*n* = 3, ^***^ *p* < 0.001). **Figure S3**. Signaling pathway analysis of enriched genes in 3D conditions. (a) Heatmaps of 3D enriched genes belonging to the following GO classes: extracellular matrix, vasculature development, regeneration, response to steroid hormone, response to wounding, and regulation to cell proliferation. (b) Q-PCR validation of representative endothelial marker genes enriched in 3D GM differentiation in iPSCs (*n* = 3, ^**^ *p* < 0.005, ^***^ *p* < 0.001, 3D versus 2D; *t* test), related to Fig. 5. **Figure S4**. In vivo tumorigenicity test of MESP1^+^ cells. (a) Representative images showing teratoma formation in the right leg of the mouse injected with undifferentiated MESP1-mTomato cells (*red circle*). No teratoma was found in immune-deficient mice injected with the same number of differentiated MESP1-mTomato^+^ cells. (b) Table summarizing the teratoma formation result from undifferentiated MESP1-mTomato and differentiated MESP1-mTomato^+^ cells. Similar results were obtained with human iPSCs and other ESC lines (H1 and H7) (data not shown). (ZIP 10253 kb)
Additional file 2: Table S2. Excel tables for genes significantly upregulated in MESP1^+^ cells compared with undifferentiated hESCs and genes significantly downregulated in MESP1^−^ cells compared with undifferentiated hESCs. (XLSX 53 kb)
Additional file 3: Table S3. Gene ontology classes overrepresented in day 3 MESP1-mTomato-positive cells versus day 3 MESP1-mTomato-negative cells, *p* < 0.05. (DOCX 19 kb)
Additional file 6: Table S4. Gene ontology classes overrepresented in MESP1^+^ cells differentiated in 3D microniches compared with cells differentiated in 2D culture condition for 5 days, *p* < 0.05. (DOCX 20 kb)

## Abbreviations

2D: two-dimensional
3D: three-dimensional
bFGF: basic fibroblast growth factor
bHLH: basic helix–loop–helix
BMP: bone morphogenetic protein
cDNA: complementary DNA
CLI: critical limb ischemia
DAPI: 4′, 6-diamidino-2-phenylindole
DAVID: database for annotation, visualization, and integrated discovery
DMEM: Dulbecco’s modified Eagle’s medium
EB: embryoid bodies
EC: endothelial cells
ECM: extracellular matrix
FACS: fluorescence-activated cell sorting
GM: gelatin microcryogels
GO: gene ontology
hESC: human embryonic stem cell
hiPSC: human induced pluripotent stem cell
hPSC: human pluripotent stem cell
MESP1: mesoderm posterior 1
PCR: polymerase chain reaction
Q-PCR: real-time quantitative polymerase chain reaction
ROCK: Rho-associated coiled-coil kinase
S1P: Sphingosine-1-phosphate
S1PR: Sphingosine-1-phosphate receptor
VEGF: vascular endothelial growth factor
