# Supplementary material for: Optimizing mesoderm progenitor selection and three-dimensional microniche culture allows highly efficient endothelial differentiation and ischemic tissue repair from human pluripotent stem cells
Source: Stem Cell Res Ther. 2017 Jan 23;8:6. doi: 10.1186/s13287-016-0455-4 (PMC5259899; doi:10.1186/s13287-016-0455-4)
Supplement: Additional file 1: Table S1. — Primers used in this study for gene expression analysis. (DOCX 25 kb) [file 13287_2016_455_MOESM1_ESM.docx]

Table S1. Primers for Q-PCR

| Gene | Accession No. | Forward primer | Reverse primer | T. |
| --- | --- | --- | --- | --- |
| *GAPDH* | NM_002046 | tgatgacatcaagaaggtggtgaag | tccttggaggccatgtgggccat | 60 |
| *OCT4* | NM_002701 | cgaccatctgccgctttgag | ccccctgtcccccattccta | 60 |
| *SOX2* | NM_003106 | cccccggcggcaatagca | tcggcgccggggagatacat | 60 |
| *NANOG* | NM_024865 | ggatggtctcgatctcctga | cctcccaatcccaaacaata | 60 |
| *T* | NM_003181 | gtccacctgcaaatcctcat | cacaggctggggtactgact | 60 |
| *MESP1* | NM_018670 | cgctgtgccccgacgact | ggcatccaggtctccaacag | 60 |
| *KDR* | NM_002253 | tgagcaaagggtggaggtgact | cttgcacaaagtgacacgttgag | 60 |
| *CXCR4* | NM_003467 | cgtggaacgtttttcctgtt | ggtgctgaaatcaacccact | 60 |
| *GATA4* | NM_002052 | gcatcaaccggccgctcatca | ggttcttgggcttccgttttct | 60 |
| *TBX5* | NM_000192 | ccaggagcatagccaaatttac | agggcttcttatagggatggtc | 60 |
| *NKX2.5* | NM_004387 | aaggaccctagagccgaaaag | gccgctccagctcatagacc | 60 |
| *SOX17* | NM_022454 | ctgccacttgaacagtttgg | gaggaagctgttttgggaca | 60 |
| *PAX6* | NM_000280 | cacggtccaatcattttgtg | aaggctttggcatggttttt | 60 |
| *S1P1* | NM_001400 | ggctctccgaacgcaacttc | cgctactccagacgaacgct | 60 |
| *S1P2* | NM_004230 | gcctagccagttctgaaagc | tgcccagaaacaggtacattg | 60 |
| *S1P3* | NM_005226 | cctgcgggagcattaccagt | gcagagagccaggttgccaa | 60 |
| *CD31* | NM_000442 | ggtggatgaggtccagatttc | cagcacaatgtcctctccag | 60 |
| *FOXC1* | NM_001453 | tgcttttcagagacctgcttt | gcaaggaagaaggcaagaga | 60 |
| *FLT1* | NM_002019 | cagcatacctcactgttcaagg | ccacacaggtgcatgttagag | 60 |
| *ID1* | NM_002165 | ccagaaccgcaaggtgag | ggtccctgatgtagtcgatga | 60 |
| *TAL1* | NM_003189 | ctatgagatggagattactgatggtc | gtgtggggatcagcttgc | 60 |
| *CDH5* | NM_001795 | gaccgggagaatatctcagagt | cattgaacaaccgatgcgtga | 60 |
| *CD34* | NM_001773 | gcgctttgcttgctgagt | gggtagcagtaccgttgttgt | 60 |
| *ETV2* | NM_014209 | ccaaggggacacgccgacag | gaccacggaagagcctgagag | 60 |
| *GATA2* | NM_032638 | cgaggagctgtcaaagtgc | acaggtgccatgtgtccag | 60 |
| *BMP7* | NM_001719 | accactgggtggtcaatcc | caacttggggttgatgctct | 60 |
| *TGFB1* | NM_000660 | actactacgccaaggaggtcac | tgcttgaacttgtcatagatttcg | 60 |
