# Supplementary figures and images for: Optimizing mesoderm progenitor selection and three-dimensional microniche culture allows highly efficient endothelial differentiation and ischemic tissue repair from human pluripotent stem cells
Source: Stem Cell Res Ther. 2017 Jan 23;8:6. doi: 10.1186/s13287-016-0455-4 (PMC5259899; doi:10.1186/s13287-016-0455-4)

Figure S1

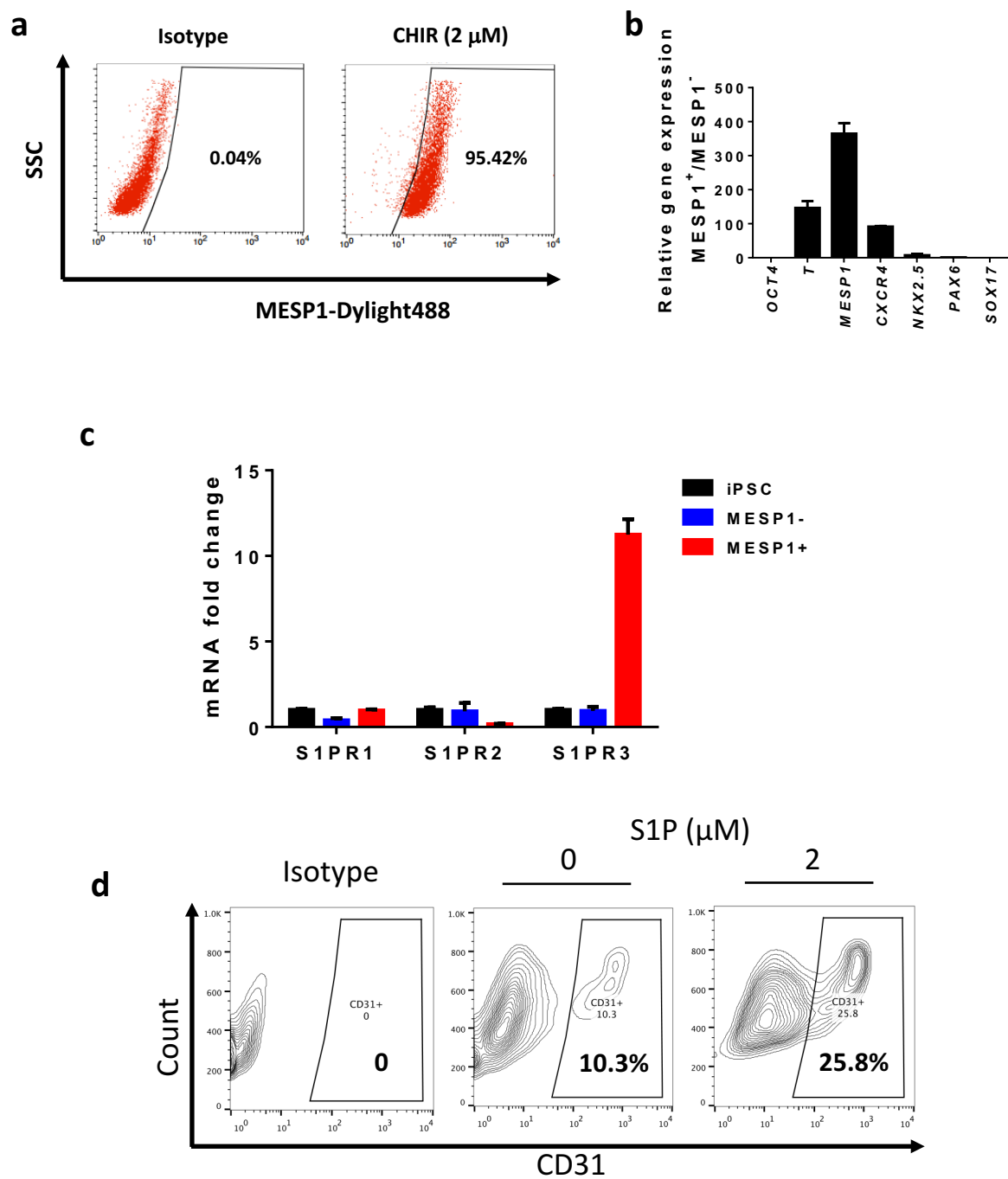

Supplement: Additional file 4: Figure S1. — Differentiation of hiPSCs to endothelial cells through an MESP1+ mesoderm progenitor stage. (a) FACS analysis showing 95.42% of cells stained positive for MESP1+ antibody after treating with CHIR99021 (2 μM) for 3 days. (b) Q-PCR analysis showing the downregulation of pluripotency, endoderm and ectoderm marker gene, and the upregulation of mesoderm and cardiac marker genes in MESP1+ cells vs. MESP1- cells (n = 3). (c) Q-PCR analysis of S1P receptor expression in undifferentiated iPSCs in differentiation day 3 MESP1+ and MESP1- cells (n = 3). (d) FACS analysis showing S1P treatment greatly enhanced the generation of CD31+ cells on differentiation day 12. (PDF 175 kb) [file 13287_2016_455_MOESM4_ESM.pdf]

Figure S3

a

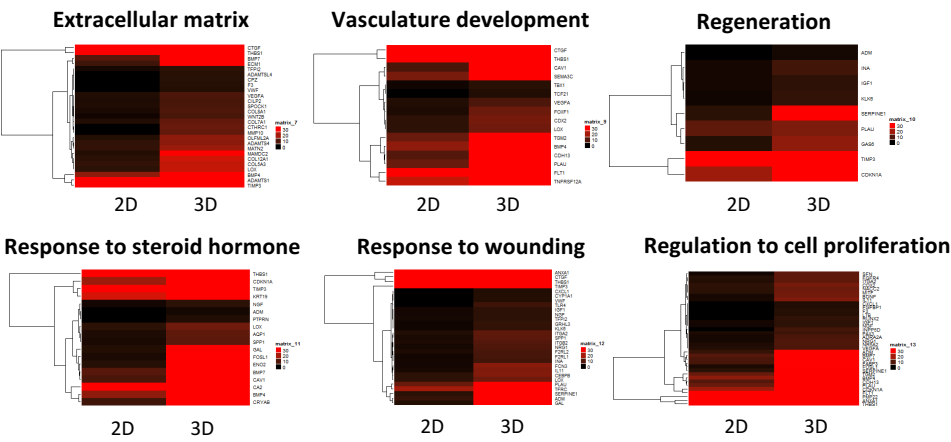

b

Representative gene expression of CD34-iPSC cultured in 2D and 3D

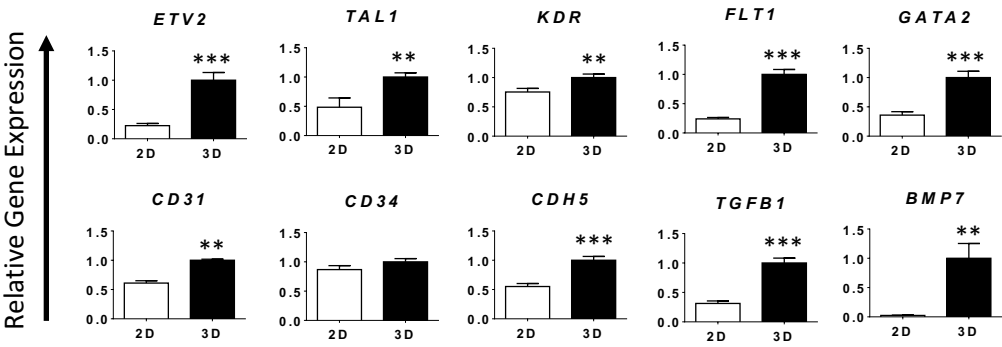

Supplement: Additional file 5: Figure S2. — 3D microniche dramatically improved endothelial differentiation efficiency from hiPSCs. (a and b) Immunostaining showing CD31 expression in 2D (a) and 3D GM (b) on differentiated day 12 respectively. Scale bars: 100 μm. (c) Percentage of CD31+ cells on differentiated day 12 in 2D and 3D GM (n = 3, *** p < 0.001). Figure S3. Signaling pathway analysis of enriched genes in 3D conditions. (a) Heatmaps of 3D enriched genes belonging to the following GO classes: extracellular matrix, vasculature development, regeneration, response to steroid hormone, response to wounding, and regulation to cell proliferation. (b) Q-PCR validation of representative endothelial marker genes enriched in 3D GM differentiation in iPSCs (n = 3, ** p < 0.005, *** p < 0.001, 3D versus 2D; t test), related to Fig. 5. Figure S4. In vivo tumorigenicity test of MESP1+ cells. (a) Representative images showing teratoma formation in the right leg of the mouse injected with undifferentiated MESP1-mTomato cells (red circle). No teratoma was found in immune-deficient mice injected with the same number of differentiated MESP1-mTomato+ cells. (b) Table summarizing the teratoma formation result from undifferentiated MESP1-mTomato and differentiated MESP1-mTomato+ cells. Similar results were obtained with human iPSCs and other ESC lines (H1 and H7) (data not shown). (ZIP 10253 kb) [file 13287_2016_455_MOESM5_ESM.zip › Endothelia-MicroNiche-Figure S3.pdf]

Figure S2

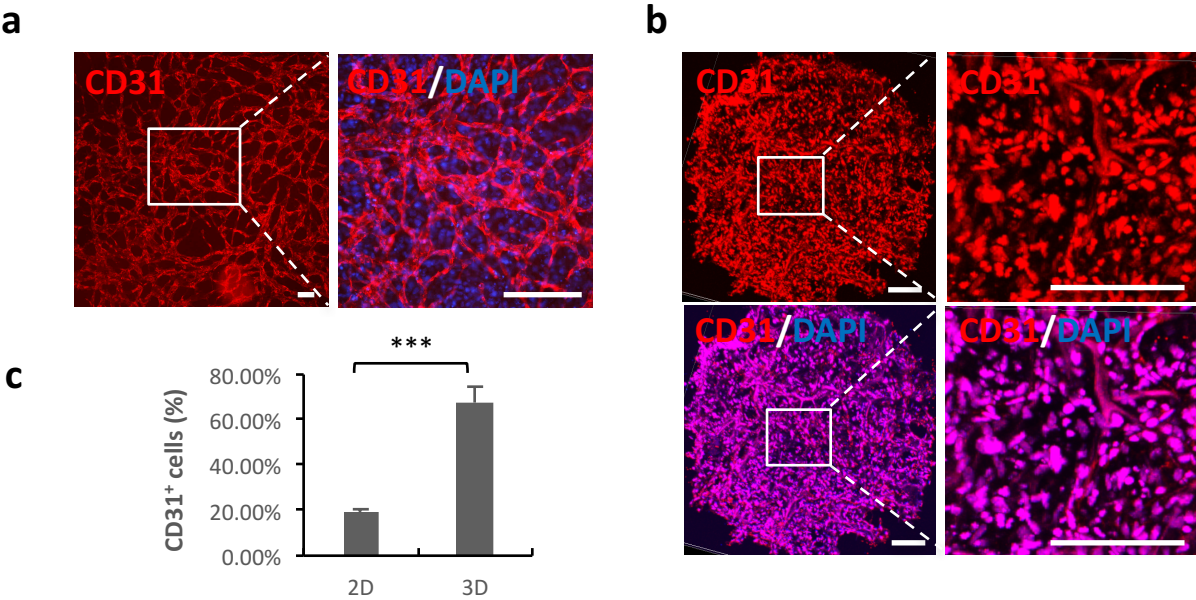

Supplement: Additional file 5: Figure S2. — 3D microniche dramatically improved endothelial differentiation efficiency from hiPSCs. (a and b) Immunostaining showing CD31 expression in 2D (a) and 3D GM (b) on differentiated day 12 respectively. Scale bars: 100 μm. (c) Percentage of CD31+ cells on differentiated day 12 in 2D and 3D GM (n = 3, *** p < 0.001). Figure S3. Signaling pathway analysis of enriched genes in 3D conditions. (a) Heatmaps of 3D enriched genes belonging to the following GO classes: extracellular matrix, vasculature development, regeneration, response to steroid hormone, response to wounding, and regulation to cell proliferation. (b) Q-PCR validation of representative endothelial marker genes enriched in 3D GM differentiation in iPSCs (n = 3, ** p < 0.005, *** p < 0.001, 3D versus 2D; t test), related to Fig. 5. Figure S4. In vivo tumorigenicity test of MESP1+ cells. (a) Representative images showing teratoma formation in the right leg of the mouse injected with undifferentiated MESP1-mTomato cells (red circle). No teratoma was found in immune-deficient mice injected with the same number of differentiated MESP1-mTomato+ cells. (b) Table summarizing the teratoma formation result from undifferentiated MESP1-mTomato and differentiated MESP1-mTomato+ cells. Similar results were obtained with human iPSCs and other ESC lines (H1 and H7) (data not shown). (ZIP 10253 kb) [file 13287_2016_455_MOESM5_ESM.zip › Endothelia-MicroNiche-Figure S2.pdf]
