# Supplementary material for: Optimizing mesoderm progenitor selection and three-dimensional microniche culture allows highly efficient endothelial differentiation and ischemic tissue repair from human pluripotent stem cells
Source: Stem Cell Res Ther. 2017 Jan 23;8:6. doi: 10.1186/s13287-016-0455-4 (PMC5259899; doi:10.1186/s13287-016-0455-4)
Supplement: Additional file 3: Table S3. — Gene ontology classes overrepresented in day 3 MESP1-mTomato-positive cells versus day 3 MESP1-mTomato-negative cells, p < 0.05. (DOCX 19 kb) [file 13287_2016_455_MOESM3_ESM.docx]

**Table S3. Gene ontology classes overrepresented in day 3 MESP1-mTomato positive cells versus day 3 MESP1-mTomato negative cells (LogFC>1.0, P<0.01).**

| GO Accession | GO Term Name | P-value |
| --- | --- | --- |
| GO:0048598 | embryonic morphogenesis | 2.1E-09 |
| GO:0003002 | regionalization | 2.4E-09 |
| GO:0007389 | pattern specification process | 6.7E-09 |
| GO:0009792 | embryo development ending in birth or egg hatching | 9.4E-09 |
| GO:0035107 | appendage morphogenesis | 1.3E-08 |
| GO:0035108 | limb morphogenesis | 1.3E-08 |
| GO:0048736 | appendage development | 2.1E-08 |
| GO:0060173 | limb development | 2.1E-08 |
| GO:0001501 | skeletal system development | 2.3E-08 |
| GO:0035113 | embryonic appendage morphogenesis | 3.5E-08 |
| GO:0030326 | embryonic limb morphogenesis | 3.5E-08 |
| GO:0043009 | chordate embryonic development | 4.2E-08 |
| GO:0009952 | anterior/posterior pattern specification | 8.3E-08 |
| GO:0048706 | embryonic skeletal system development | 1.2E-06 |
| GO:0048568 | embryonic organ development | 2.5E-06 |
| GO:0048705 | skeletal system morphogenesis | 3.9E-06 |
| GO:0048568 | embryonic organ development | 3.1E-05 |
| GO:0048598 | embryonic morphogenesis | 3.3E-05 |
| GO:0009952 | anterior/posterior pattern specification | 3.5E-05 |
| GO:0048568 | embryonic organ development | 3.9E-05 |
| GO:0048729 | tissue morphogenesis | 4.7E-05 |
| GO:0043565 | sequence-specific DNA binding | 4.8E-05 |
| GO:0005887 | integral component of plasma membrane | 7.9E-05 |
| GO:0046914 | transition metal ion binding | 1.0E-04 |
| GO:0048562 | embryonic organ morphogenesis | 1.1E-04 |
| GO:0031226 | intrinsic component of plasma membrane | 1.1E-04 |
| GO:0048598 | embryonic morphogenesis | 1.2E-04 |
| GO:0007389 | pattern specification process | 1.3E-04 |
| GO:0003002 | regionalization | 1.3E-04 |
| GO:0048704 | embryonic skeletal system morphogenesis | 1.4E-04 |
| GO:0043235 | receptor complex | 1.5E-04 |
| GO:0007389 | pattern specification process | 1.5E-04 |
| GO:0003700 | transcription factor activity, sequence-specific DNA binding | 1.6E-04 |
| GO:0005887 | integral component of plasma membrane | 1.7E-04 |
| GO:0008083 | growth factor activity | 2.1E-04 |
| GO:0008284 | positive regulation of cell proliferation | 2.5E-04 |
| GO:0031226 | intrinsic component of plasma membrane | 2.6E-04 |
| GO:0042127 | regulation of cell proliferation | 2.8E-04 |
