# Supplementary material for: Optimizing mesoderm progenitor selection and three-dimensional microniche culture allows highly efficient endothelial differentiation and ischemic tissue repair from human pluripotent stem cells
Source: Stem Cell Res Ther. 2017 Jan 23;8:6. doi: 10.1186/s13287-016-0455-4 (PMC5259899; doi:10.1186/s13287-016-0455-4)
Supplement: Additional file 6: Table S4. — Gene ontology classes overrepresented in MESP1+ cells differentiated in 3D microniches compared with cells differentiated in 2D culture condition for 5 days, p < 0.05. (DOCX 20 kb) [file 13287_2016_455_MOESM6_ESM.docx]

**Table S4. Gene ontology classes overrepresented in day 8 MESP1^+^ cells differentiated in 3D microniches vs day 8 MESP1^+^ cells differentiated in 2D culture system. (****LogFC>1.0, P<0.01).**

| GO Accession | GO Term Name | P-value |
| --- | --- | --- |
| GO:0005576 | extracellular region | 3.8E-17 |
| GO:0044421 | extracellular region part | 4.8E-16 |
| GO:0031012 | extracellular matrix | 8.0E-13 |
| GO:0005578 | proteinaceous extracellular matrix | 3.8E-11 |
| GO:0042127 | regulation of cell proliferation | 1.8E-09 |
| GO:0005615 | extracellular space | 2.1E-08 |
| GO:0008083 | growth factor activity | 8.3E-08 |
| GO:0007267 | cell-cell signaling | 1.3E-07 |
| GO:0009628 | response to abiotic stimulus | 8.3E-07 |
| GO:0008284 | positive regulation of cell proliferation | 9.3E-07 |
| GO:0009719 | response to endogenous stimulus | 1.8E-06 |
| GO:0040008 | regulation of growth | 2.0E-06 |
| GO:0044459 | plasma membrane part | 2.4E-06 |
| GO:0009611 | response to wounding | 2.7E-06 |
| GO:0048545 | response to steroid hormone stimulus | 4.7E-06 |
| GO:0032101 | regulation of response to external stimulus | 6.4E-06 |
| GO:0009725 | response to hormone stimulus | 7.5E-06 |
| GO:0005887 | integral to plasma membrane | 9.0E-06 |
| GO:0031226 | intrinsic to plasma membrane | 9.1E-06 |
| GO:0051240 | positive regulation of multicellular organismal process | 1.1E-05 |
| GO:0044057 | regulation of system process | 1.2E-05 |
| GO:0042802 | identical protein binding | 1.3E-05 |
| GO:0009266 | response to temperature stimulus | 1.4E-05 |
| GO:0007155 | cell adhesion | 1.5E-05 |
| GO:0022610 | biological adhesion | 1.5E-05 |
| GO:0001558 | regulation of cell growth | 2.1E-05 |
| GO:0045785 | positive regulation of cell adhesion | 3.1E-05 |
| GO:0009968 | negative regulation of signal transduction | 3.2E-05 |
| GO:0005179 | hormone activity | 3.8E-05 |
| GO:0008544 | epidermis development | 4.0E-05 |
| GO:0006916 | anti-apoptosis | 4.5E-05 |
| GO:0042803 | protein homodimerization activity | 4.6E-05 |
| GO:0001944 | vasculature development | 5.4E-05 |
| GO:0009967 | positive regulation of signal transduction | 5.4E-05 |
| GO:0010033 | response to organic substance | 6.3E-05 |
| GO:0007166 | cell surface receptor linked signal transduction | 6.5E-05 |
| GO:0046983 | protein dimerization activity | 8.5E-05 |
| GO:0030155 | regulation of cell adhesion | 8.7E-05 |
| GO:0010941 | regulation of cell death | 9.1E-05 |
| GO:0010647 | positive regulation of cell communication | 9.1E-05 |
| GO:0031099 | regeneration | 9.5E-05 |
| GO:0043066 | negative regulation of apoptosis | 9.7E-05 |
| GO:0009991 | response to extracellular stimulus | 1.0E-04 |
| GO:0035295 | tube development | 1.0E-04 |
| GO:0007398 | ectoderm development | 1.0E-04 |
| GO:0043069 | negative regulation of programmed cell death | 1.2E-04 |
| GO:0048771 | tissue remodeling | 1.2E-04 |
| GO:0001568 | blood vessel development | 1.2E-04 |
| GO:0043627 | response to estrogen stimulus | 1.3E-04 |
| GO:0060548 | negative regulation of cell death | 1.3E-04 |
| GO:0008285 | negative regulation of cell proliferation | 1.3E-04 |
| GO:0042445 | hormone metabolic process | 1.4E-04 |
| GO:0042981 | regulation of apoptosis | 1.4E-04 |
| GO:0010648 | negative regulation of cell communication | 1.4E-04 |
| GO:0016265 | death | 1.5E-04 |
| GO:0050920 | regulation of chemotaxis | 1.6E-04 |
| GO:0002685 | regulation of leukocyte migration | 1.6E-04 |
| GO:0001763 | morphogenesis of a branching structure | 1.6E-04 |
| GO:0007498 | mesoderm development | 1.6E-04 |
| GO:0043067 | regulation of programmed cell death | 1.7E-04 |
| GO:0005886 | plasma membrane | 1.8E-04 |
| GO:0048514 | blood vessel morphogenesis | 2.0E-04 |
| GO:0048660 | regulation of smooth muscle cell proliferation | 2.2E-04 |
| GO:0042060 | wound healing | 2.2E-04 |
| GO:0010817 | regulation of hormone levels | 2.3E-04 |
| GO:0042493 | response to drug | 2.6E-04 |
| GO:0031644 | regulation of neurological system process | 2.6E-04 |
| GO:0008219 | cell death | 2.7E-04 |
| GO:0012501 | programmed cell death | 2.8E-04 |
| GO:0009891 | positive regulation of biosynthetic process | 3.0E-04 |
| GO:0005125 | cytokine activity | 3.0E-04 |
| GO:0032103 | positive regulation of response to external stimulus | 3.1E-04 |
| GO:0005184 | neuropeptide hormone activity | 3.3E-04 |
| GO:0048754 | branching morphogenesis of a tube | 3.5E-04 |
| GO:0048168 | regulation of neuronal synaptic plasticity | 3.7E-04 |
| GO:0030193 | regulation of blood coagulation | 3.7E-04 |
| GO:0033273 | response to vitamin | 3.9E-04 |
| GO:0007584 | response to nutrient | 4.2E-04 |
| GO:0010627 | regulation of protein kinase cascade | 4.4E-04 |
| GO:0070482 | response to oxygen levels | 4.5E-04 |
| GO:0006915 | apoptosis | 4.5E-04 |
| GO:0060429 | epithelium development | 4.6E-04 |
| GO:0031328 | positive regulation of cellular biosynthetic process | 4.7E-04 |
| GO:0045944 | positive regulation of transcription from RNA polymerase II promoter | 4.9E-04 |
| GO:0030017 | sarcomere | 4.9E-04 |
| GO:0008201 | heparin binding | 5.1E-04 |
| GO:0044420 | extracellular matrix part | 5.3E-04 |
| GO:0010628 | positive regulation of gene expression | 5.4E-04 |
| GO:0002687 | positive regulation of leukocyte migration | 5.7E-04 |
| GO:0051094 | positive regulation of developmental process | 5.7E-04 |
| GO:0007517 | muscle organ development | 6.3E-04 |
| GO:0051969 | regulation of transmission of nerve impulse | 6.5E-04 |
| GO:0030335 | positive regulation of cell migration | 6.6E-04 |
| GO:0009408 | response to heat | 7.4E-04 |
| GO:0050818 | regulation of coagulation | 7.6E-04 |
| GO:0010557 | positive regulation of macromolecule biosynthetic process | 8.8E-04 |
| GO:0031667 | response to nutrient levels | 9.5E-04 |
| GO:0007389 | pattern specification process | 9.8E-04 |
| GO:0050921 | positive regulation of chemotaxis | 9.8E-04 |
| GO:0001666 | response to hypoxia | 1.0E-03 |
| GO:0030247 | polysaccharide binding | 1.1E-03 |
| GO:0001871 | pattern binding | 1.1E-03 |
| GO:0015171 | amino acid transmembrane transporter activity | 1.1E-03 |
| GO:0050804 | regulation of synaptic transmission | 1.2E-03 |
| GO:0030855 | epithelial cell differentiation | 1.2E-03 |
| GO:0042327 | positive regulation of phosphorylation | 1.2E-03 |
| GO:0045893 | positive regulation of transcription, DNA-dependent | 1.3E-03 |
| GO:0030016 | myofibril | 1.3E-03 |
| GO:0006357 | regulation of transcription from RNA polymerase II promoter | 1.3E-03 |
| GO:0031589 | cell-substrate adhesion | 1.3E-03 |
| GO:0051272 | positive regulation of cell motion | 1.3E-03 |
| GO:0040017 | positive regulation of locomotion | 1.3E-03 |
| GO:0048661 | positive regulation of smooth muscle cell proliferation | 1.3E-03 |
| GO:0010035 | response to inorganic substance | 1.4E-03 |
| GO:0045165 | cell fate commitment | 1.4E-03 |
| GO:0050795 | regulation of behavior | 1.4E-03 |
| GO:0051254 | positive regulation of RNA metabolic process | 1.4E-03 |
| GO:0045597 | positive regulation of cell differentiation | 1.4E-03 |
| GO:0008361 | regulation of cell size | 1.5E-03 |
| GO:0050927 | positive regulation of positive chemotaxis | 1.5E-03 |
| GO:0050926 | regulation of positive chemotaxis | 1.5E-03 |
| GO:0045941 | positive regulation of transcription | 1.5E-03 |
| GO:0044449 | contractile fiber part | 1.5E-03 |
| GO:0010562 | positive regulation of phosphorus metabolic process | 1.5E-03 |
| GO:0045937 | positive regulation of phosphate metabolic process | 1.5E-03 |
| GO:0002020 | protease binding | 1.5E-03 |
| GO:0042246 | tissue regeneration | 1.6E-03 |
| GO:0008237 | metallopeptidase activity | 1.6E-03 |
| GO:0048878 | chemical homeostasis | 1.6E-03 |
| GO:0005539 | glycosaminoglycan binding | 1.6E-03 |
| GO:0048167 | regulation of synaptic plasticity | 1.7E-03 |
| GO:0031982 | vesicle | 1.7E-03 |
| GO:0005604 | basement membrane | 1.7E-03 |
| GO:0030246 | carbohydrate binding | 1.8E-03 |
| GO:0008283 | cell proliferation | 1.8E-03 |
| GO:0042312 | regulation of vasodilation | 1.8E-03 |
| GO:0010740 | positive regulation of protein kinase cascade | 2.0E-03 |
| GO:0048520 | positive regulation of behavior | 2.1E-03 |
| GO:0004222 | metalloendopeptidase activity | 2.2E-03 |
| GO:0030334 | regulation of cell migration | 2.2E-03 |
| GO:0046942 | carboxylic acid transport | 2.2E-03 |
| GO:0015849 | organic acid transport | 2.3E-03 |
| GO:0001657 | ureteric bud development | 2.3E-03 |
| GO:0035239 | tube morphogenesis | 2.4E-03 |
| GO:0050730 | regulation of peptidyl-tyrosine phosphorylation | 2.4E-03 |
| GO:0048762 | mesenchymal cell differentiation | 2.4E-03 |
| GO:0014031 | mesenchymal cell development | 2.4E-03 |
| GO:0043292 | contractile fiber | 2.5E-03 |
| GO:0006928 | cell motion | 2.5E-03 |
| GO:0046660 | female sex differentiation | 2.6E-03 |
| GO:0046545 | development of primary female sexual characteristics | 2.6E-03 |
| GO:0060485 | mesenchyme development | 2.7E-03 |
| GO:0001501 | skeletal system development | 2.7E-03 |
| GO:0051173 | positive regulation of nitrogen compound metabolic process | 2.7E-03 |
| GO:0001934 | positive regulation of protein amino acid phosphorylation | 2.8E-03 |
| GO:0032403 | protein complex binding | 2.9E-03 |
| GO:0001655 | urogenital system development | 3.0E-03 |
| GO:0045926 | negative regulation of growth | 3.0E-03 |
| GO:0005581 | collagen | 3.0E-03 |
| GO:0001974 | blood vessel remodeling | 3.1E-03 |
| GO:0010604 | positive regulation of macromolecule metabolic process | 3.3E-03 |
| GO:0030308 | negative regulation of cell growth | 3.4E-03 |
| GO:0004175 | endopeptidase activity | 3.5E-03 |
| GO:0016477 | cell migration | 3.5E-03 |
| GO:0045778 | positive regulation of ossification | 3.6E-03 |
| GO:0048732 | gland development | 3.7E-03 |
| GO:0010942 | positive regulation of cell death | 3.7E-03 |
| GO:0032844 | regulation of homeostatic process | 3.8E-03 |
| GO:0009612 | response to mechanical stimulus | 3.9E-03 |
| GO:0005275 | amine transmembrane transporter activity | 4.0E-03 |
| GO:0042592 | homeostatic process | 4.1E-03 |
| GO:0051260 | protein homooligomerization | 4.2E-03 |
| GO:0000267 | cell fraction | 4.3E-03 |
| GO:0001822 | kidney development | 4.5E-03 |
| GO:0001759 | induction of an organ | 4.6E-03 |
| GO:0005198 | structural molecule activity | 4.7E-03 |
| GO:0002761 | regulation of myeloid leukocyte differentiation | 4.7E-03 |
| GO:0050878 | regulation of body fluid levels | 5.0E-03 |
| GO:0051241 | negative regulation of multicellular organismal process | 5.0E-03 |
| GO:0034754 | cellular hormone metabolic process | 5.1E-03 |
| GO:0045792 | negative regulation of cell size | 5.4E-03 |
| GO:0030324 | lung development | 5.4E-03 |
| GO:0014070 | response to organic cyclic substance | 5.6E-03 |
| GO:0042692 | muscle cell differentiation | 5.6E-03 |
| GO:0031410 | cytoplasmic vesicle | 5.7E-03 |
| GO:0045927 | positive regulation of growth | 5.9E-03 |
| GO:0050840 | extracellular matrix binding | 5.9E-03 |
| GO:0003012 | muscle system process | 6.0E-03 |
| GO:0040012 | regulation of locomotion | 6.1E-03 |
| GO:0045935 | positive regulation of nucleobase, nucleoside, nucleotide and nucleic acid metabolic process | 6.1E-03 |
| GO:0051270 | regulation of cell motion | 6.3E-03 |
| GO:0007596 | blood coagulation | 6.4E-03 |
| GO:0030323 | respiratory tube development | 6.4E-03 |
| GO:0050817 | coagulation | 6.4E-03 |
| GO:0001656 | metanephros development | 6.4E-03 |
| GO:0022602 | ovulation cycle process | 6.5E-03 |
| GO:0051174 | regulation of phosphorus metabolic process | 6.6E-03 |
| GO:0019220 | regulation of phosphate metabolic process | 6.6E-03 |
| GO:0043065 | positive regulation of apoptosis | 6.7E-03 |
| GO:0005509 | calcium ion binding | 6.8E-03 |
| GO:0031988 | membrane-bounded vesicle | 6.9E-03 |
| GO:0001525 | angiogenesis | 7.0E-03 |
| GO:0046850 | regulation of bone remodeling | 7.0E-03 |
| GO:0045124 | regulation of bone resorption | 7.0E-03 |
| GO:0032535 | regulation of cellular component size | 7.0E-03 |
| GO:0043068 | positive regulation of programmed cell death | 7.1E-03 |
| GO:0030198 | extracellular matrix organization | 7.2E-03 |
| GO:0048608 | reproductive structure development | 7.2E-03 |
| GO:0043296 | apical junction complex | 7.5E-03 |
| GO:0008585 | female gonad development | 7.5E-03 |
| GO:0048511 | rhythmic process | 8.0E-03 |
| GO:0007548 | sex differentiation | 8.0E-03 |
| GO:0019838 | growth factor binding | 8.1E-03 |
| GO:0042325 | regulation of phosphorylation | 8.2E-03 |
| GO:0010038 | response to metalion | 8.4E-03 |
| GO:0014902 | myotube differentiation | 8.5E-03 |
| GO:0050731 | positive regulation of peptidyl-tyrosine phosphorylation | 8.5E-03 |
| GO:0048638 | regulation of developmental growth | 8.5E-03 |
| GO:0006936 | muscle contraction | 8.7E-03 |
| GO:0016327 | apicolateral plasma membrane | 8.9E-03 |
| GO:0007599 | hemostasis | 9.0E-03 |
| GO:0060541 | respiratory system development | 9.0E-03 |
| GO:0044093 | positive regulation of molecular function | 9.3E-03 |
| GO:0043410 | positive regulation of MAPKKK cascade | 9.3E-03 |
| GO:0060562 | epithelial tube morphogenesis | 9.4E-03 |
| GO:0042698 | ovulation cycle | 9.4E-03 |
| GO:0051674 | localization of cell | 9.5E-03 |
| GO:0048870 | cell motility | 9.5E-03 |
| GO:0005626 | insoluble fraction | 9.8E-03 |
